# Serum Metabolomic Profiling to Reveal Potential Biomarkers for the Diagnosis of Fatty Liver Hemorrhagic Syndrome in Laying Hens

**DOI:** 10.3389/fphys.2021.590638

**Published:** 2021-02-09

**Authors:** Lianying Guo, Jun Kuang, Yu Zhuang, Jialin Jiang, Yan Shi, Cheng Huang, Changming Zhou, Puzhi Xu, Ping Liu, Cong Wu, Guoliang Hu, Xiaoquan Guo

**Affiliations:** ^1^Jiangxi Provincial Key Laboratory for Animal Health, College of Animal Science and Technology, Jiangxi Agricultural University, Nanchang, China; ^2^Jiangxi Biological Vocational College, Nanchang University, Nanchang, China; ^3^School of Computer and Information Engineering, Jiangxi Agricultural University, Nanchang, China

**Keywords:** fatty liver hemorrhage syndrome, serum, gas chromatography time-of-flight mass spectrometry, diagnosis, biomarkers, metabolomics

## Abstract

Fatty liver hemorrhage syndrome (FLHS), a nutritional and metabolic disease that frequently occurs in laying hens, causes serious losses to the poultry industry. Nowadays, the traditional clinical diagnosis of FLHS still has its limitations. Therefore, searching for some metabolic biomarkers and elucidating the metabolic pathway *in vivo* are useful for the diagnosis and prevention of FLHS. In the present study, a model of FLHS in laying hens induced by feeding a high-energy, low-protein diet was established. Gas chromatography time-of-flight mass spectrometry (GC-TOF-MS) was used to analyze the metabolites in serum at days 40 and 80. The result showed that, in total, 40 differential metabolites closely related to the occurrence and development of FLHS were screened and identified, which were mainly associated with lipid metabolism, amino acid metabolism, and energy metabolism pathway disorders. Further investigation of differential metabolites showed 10 potential biomarkers such as 3-hydroxybutyric acid, oleic acid, palmitoleic acid, and glutamate were possessed of high diagnostic values by analyzing receiver operating characteristic (ROC) curves. In conclusion, this study showed that the metabolomic method based on GC-TOF-MS can be used in the clinical diagnosis of FLHS in laying hens and provide potential biomarkers for early risk evaluation of FLHS and further insights into FLHS development.

## Introduction

Fatty liver hemorrhage syndrome (FLHS) is a hepatic manifestation of nutritional and metabolic disease, which is characterized by liver bleeding, fat accumulation, and sudden death caused by lipid metabolic disorders (Butler, 1976; Jensen et al., 1976; Gao et al., 2019). Nutritional, genetic, endocrine, environmental, and toxicological factors are related to the occurrence of FLHS (Squires and Leeson, 1988; Hansen and Walzem, 1993). Among them, the nutritional factor is regarded as an important cause of FLHS occurrence in production practice (Cherian et al., 2002). With the improvement in inbreeding technology and the application of cage feeding technology of commercial laying hens, FLHS has been widely observed in breeding production (Schumann et al., 2000; Trott et al., 2014). According to previous research, FLHS gave rise to a mortality rate as high as 5% in one laying cycle (Trott et al., 2014). Recently studies showed that the natural cases of FLHS often occur in nutritionally over-conditioned laying hens (Shini et al., 2019). Pathological examination showed a rich reserve of body cavity fat and an enlarged, pale, yellowish-brown to yellow, fragile liver, accompanied by subcapsular and parenchymal hematoma and bleeding (Squires and Leeson, 1988; Hansen and Walzem, 1993). However, the onset of FLHS is characterized of high concealment and slow recovery, which causes huge economic losses to the breeding industry. Therefore, it is important to make an early diagnosis of FLHS in laying hens to improve clinical treatment outcomes and reduce mortality.

Many screening methods have been used for the diagnosis and detection of FLHS in laying hens, such as biochemical analysis of serum or plasma, liver histopathological evaluation, and the related method of molecular determinants. However, there are some limitations of these methods, which impede the achievement of early monitoring. In recent years, metabolomics, as a new technique, has been used to investigate and describe the overall changes of endogenous small molecular metabolites caused by internal and external stimuli at a specific time and under specific conditions (Zira et al., 2013; Yu et al., 2020). It has been widely used in the diagnosis and monitoring of disease progression in the biomedicine field, which provides an important insight into the pathogenesis of the disease (Dettmer et al., 2007; Zhao et al., 2016). Mass spectrometry (MS) and nuclear magnetic resonance (NMR) spectroscopy are two major analytical platforms of metabolomics (Patel and Ahmed, 2015). In the metabolomics based on MS, gas chromatography time-of-flight mass spectrometry (GC-TOF-MS) is considered one of the best analytical techniques because of its powerful separation efficiency and detection sensitivity (Xiong et al., 2015). Recently, this technique has been used by researchers to investigate biomarkers related to diagnosis and potential diseases (Zhang et al., 2015; Ismail et al., 2020). For example, Chang et al. (2015) identified a set of biomarkers (succinate, inosine, and phenylalanine) that might be of great significance for the diagnosis of asthma. Rodríguez-Gallego et al. (2015) found that α-ketoglutaric acid can be used as a potential biomarker of non-alcoholic fatty liver in obese patients. At present, most of the metabolomic studies are focused on mammals, and little is known about metabolomics of laying hens. Therefore, the GC-TOF-MS technique may provide a new perspective for studying the potential metabolic response of FLHS induced by high-energy, low-protein diets in laying hens, better understand its biological mechanism, and provide potential biomarkers for its diagnosis.

In this study, we used non-targeted metabolomics technology based on GC-TOF-MS approach to detect the changes of serum metabolites in FLHS laying hens in different periods (40 and 80 days) induced by high-energy, low-protein diets. The present study aims to provide potential biomarkers for the diagnosis of FLHS in laying hens. These results could help guide the development of an objective diagnostic method for FLHS and provide insights into FLHS.

## Materials and Methods

### Experimental Design

All the experimental protocols and methods used in this study were approved by the Institutional Animal Care and Use Committee (Jiangxi Agricultural University, Nanchang, Jiangxi, China). A total of 100 15-week-old Hy-Line Brown laying hens were purchased from a local commercial pullet farm (Guohua Co., Ltd., Nanchang, Jiangxi, China). Subsequently, these 100 healthy layer hens were randomly divided into two experimental groups: the control group fed with basal diet (10 chickens per subgroup) and the disease group fed with a high-energy, low-protein diet (10 chickens per subgroup). The hens were raised in ladder cages. After about 2 weeks of acclimation, layers in the formal experiment were 120 days old. According to the Nutrient Requirement of Poultry (Dale, 1994). The dietary formula was based on published research in this laboratory (Gao et al., 2019). All layers were maintained with *ad libitum* access to food and water. The light time was according to the standard light procedure of commercial laying hens, 16L:8D (Lighting was controlled daily between 6 a.m. and 10 p.m.). The whole experimental period lasted 80 days.

The chicken flock were fasted for 12 h before on the 40th (marked as A) and 80th days (marked as B) of the formal experiment. Two hens were randomly selected from each subgroup to collect blood specimens from the left-wing vein and then euthanized by carbon inhalation. After the cervical vertebrae of the chicken were dislocated, the liver tissue was quickly separated from the body in a sterile environment and fixed in a 4% paraformaldehyde solution. The whole blood was coagulated in a tube at room temperature and centrifugated at 3,500 rpm for 10 min at 4°C. The serum samples were separated and stored at −80°C. Among the 40-day and 80-day serum samples, seven biological replicates of serum samples were randomly selected from each group, which were a total of 28 samples used for GC-TOF-MS analysis.

### Histopathological Examination of the Liver

The isolated liver tissues were put in a 4% paraformaldehyde solution at room temperature for 24 h and then treated routinely (Li et al., 2014). H&E staining was performed to evaluate the severity of liver steatosis. Then, the liver sections per chicken were examined and photographed under an optical microscope.

### Serum Sample Preparation and Gas Chromatography Time-of-Flight Mass Spectrometry Analysis

The serum sample preparation procedure and GC-TOF-MS analysis were similar to the previously published procedure with slight modification (Gu et al., 2015; Geng et al., 2020; Yu et al., 2020). In brief, take 50 μl of sample and mix it with 5 μl of internal standard (L-2-chlorophenylalanine) into 1.5-ml Eppendorf (EP) tubes, and 200 μl of methanol was added. Treat with ultrasound for 10 min (incubated in ice water). Centrifuge for 15 min at 12,000 rpm and 4°C. Transfer the supernatant of 180 μl into fresh 1.5-ml EP tubes. Take 20 μl from each sample and pool as QC sample. The dried samples (in a vacuum concentrator) were then incubated with 30 μl of methoxy amination hydrochloride at 80°C for 30 min. Subsequently, 40 μl of the bis (trimethylsilyl)trifluoroacetamide (BSTFA) reagent was added to the sample aliquots and incubated for 1.5 h at 70°C. Then, 5 μl of fatty acid methyl esters (FAMEs) (in chloroform) was added to the QC sample at room temperature. Finally, an Agilent 7890 gas chromatograph system coupled with a Pegasus HT time-of-flight mass spectrometer was used to perform GC-TOF-MS analysis. The energy was −70 eV in electron impact mode. After 4.7 min of solvent delay, mass spectrometry data were obtained at full-scan mode (m/z 50–500) at a rate of 12.5 spectra per second.

### Statistical and Data Analysis

The method described by Kind et al. (2009) was used to analyze the raw data of GC-TOF-MS. First, Chroma TOF4.3X software (LECO Corporation) and LECO-Fiehn Rtx5 database were used for compound detection, peak alignment, identification, deconvolution analysis, and integration of the peak area (Dunn et al., 2011). The SIMCA-P14.1 software package (Umetrics, Umea, Sweden) was then applied to make further data analysis, including principal component analysis (PCA) and orthogonal partial least-squares discriminant analysis (OPLS-DA). Finally, the OPLS-DA model was employed, and the variable importance in the projection (VIP) values (VIP > 1) combined with the Student’s *t*-test (*P* < 0.05) were used to define significantly differential metabolites, i.e., metabolites with significant difference in concentration, between the two groups. In addition, commercial databases including the Kyoto Encyclopedia of Genes and Genomes (KEGG^1^ /) and Bovine Metabolome Database (BMDB) were employed to further search for metabolite pathways associated with these significantly differential metabolites. MetaboAnalyst^2^ was used for pathway enrichment analysis.

The significantly differential metabolites with similarity scores over 700 in comparison with the standards were regarded as candidate biomarkers to be used for subsequent analysis. Area under the curve (AUC) and receiver operating characteristic (ROC) curve analyses were performed to determine the optimal potential biomarker for the diagnosis of FLHS. The decision criteria are as follows: the AUC of 0.9–1.0 indicated excellent performance; 0.8–0.89, good performance; 0.7–0.79, fair performance; 0.6–0.69, poor performance; and < 0.6, insignificant value (Haase-Fielitz et al., 2009). ROC curves and the area under the ROC curve (AUC) were computed using the “pROC” package in R (Robin et al., 2011).

## Results

### Pathological and Histopathological Changes of the Liver Tissue

**FIGURE 1:**
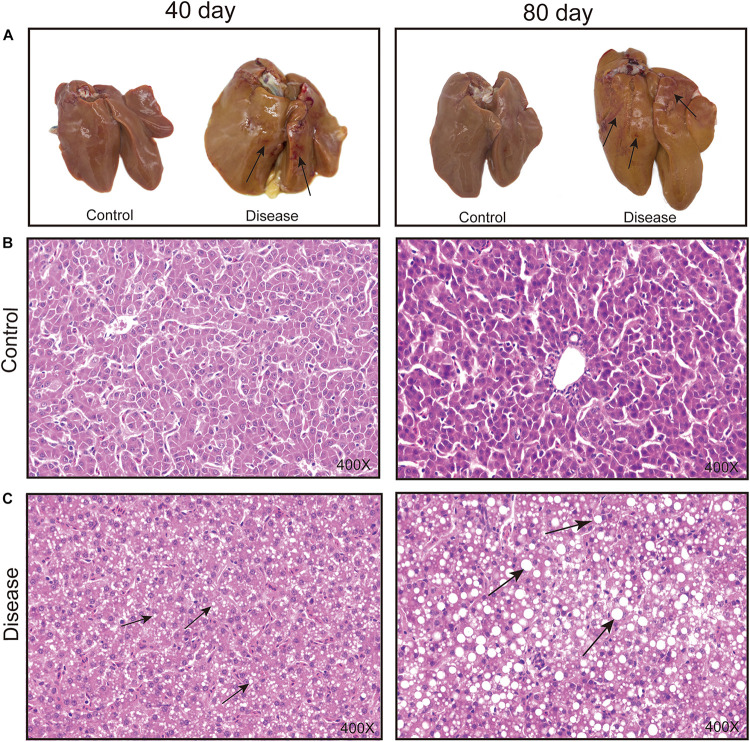
Effects of a high-energy, low-protein diet on hepatic tissue pathological change at different time points after induction. **(A)** Liver morphology alteration. The black arrowhead shows the bleeding spots and bleeding areas. **(B,C)** Histopathological changes in the liver of laying hens fed a high-energy, low-protein diet for 40 and 80 days (H&E staining). The black arrowhead shows the fat droplets.

The macroscopic liver appearance of layers from the disease group shows obvious grayish-yellow, hepatomegaly, brittleness, bleeding spots, and bleeding areas with obvious histopathological alteration over time compared with the appearance of layers from the control group (Figure 1). Meanwhile, the accumulation of lipid droplets and fat vacuoles in the liver sections of the laying hens in the disease group gradually increased, whereas the hepatocyte structure is clear and without fat vacuoles in the liver section of layers from the control group. These results were consistent with the results of previous studies (Rozenboim et al., 2016) and suggest that a high-energy, low-protein diet leads to the progressive increase in fat accumulation in hepatic tissue cells, which eventually causes FLHS.

### Metabolite Profiling Using Gas Chromatography Time-of-Flight Mass Spectrometry

Serum metabolomics based on GC-TOF-MS method was applied to further investigate the pathophysiological metabolic mechanism of the high-energy, low-protein diet-induced FLHS at different time points (40 and 80 days). A total of 686 metabolite peaks were extracted by GC-TOF-MS total ion current (TIC) chromatograms. Among them, 463 effective metabolite peaks were identified after filtering and denoising. The TIC of the serum samples from the control and disease group at days 40 and 80 is shown in Supplementary Figure 1.

### Principal Component Analysis of Serum Samples

**FIGURE 2:**
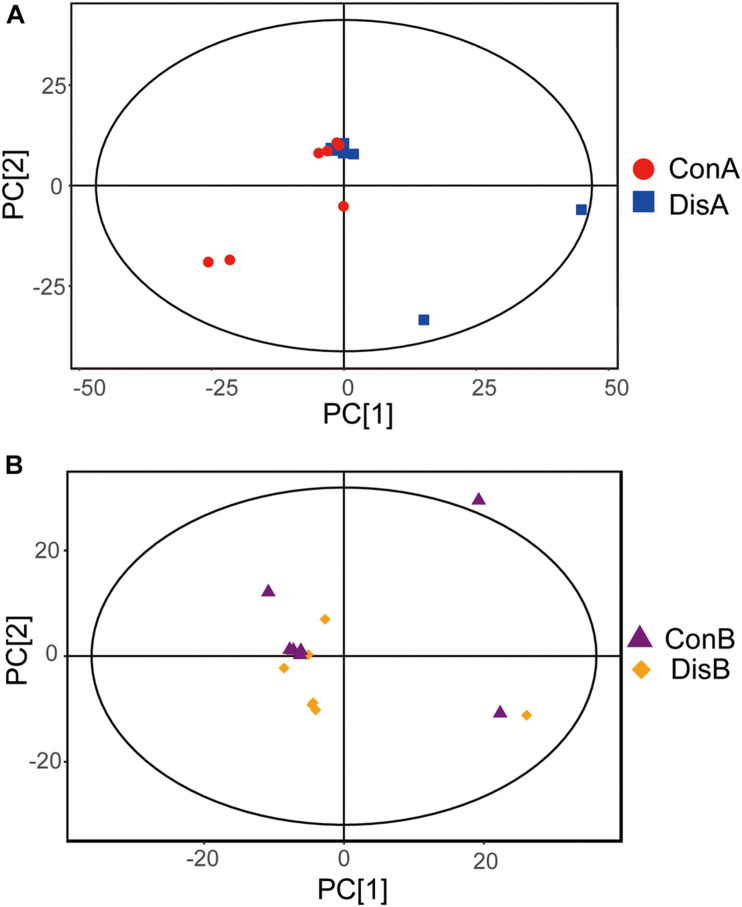
Principal component analysis (PCA) plots based on the gas chromatography time-of-flight mass spectrometry (GC-TOF-MS) analyses of serum samples from the control and disease groups laying hens after **(A)** 40 and **(B)** 80 days of feeding.

The PCA plot was employed to discriminate the different metabolic profiles and general trends affecting the FLHS laying hens at each time point (Feng et al., 2013). Of the 28 serum samples in the scoring chart for the two time points, the samples were basically within the 95% Hotelling’s *T*-square ellipse (Figure 2). The PCA score plot showed that there was no clear separation between the disease and control groups. At day 80 and in the control group, one outlier went beyond the 95% Hotelling’s *T-*square ellipse (Figure 2B), which may be caused by individual differences between individual samples.

### Orthogonal Partial Least-Squares Discriminant Analysis of Serum Samples

**FIGURE 3:**
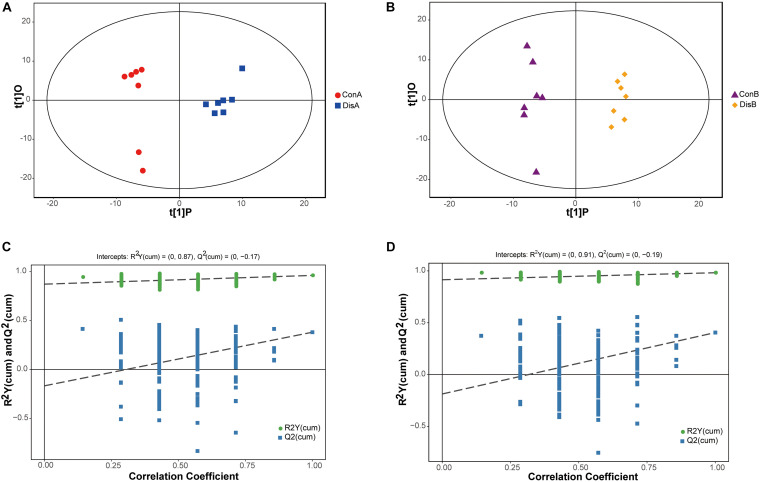
Orthogonal partial least-squares discriminant analysis (OPLS-DA) score plots **(A,B)** and corresponding validation plots of the permutation tests (200 times) of the OPLS-DA models **(C,D)**. Panels **(A,C)** are for 40 days, and panels **(B,D)** are for 80 days.

A supervised OPLS-DA model was constructed to further investigate and analyze the separation of the normal group and the disease group (Trygg et al., 2007). As shown in Figure 3, the control group and the disease group were clearly separated at the two time points (days 40 and 80). All serum samples in the score plots were also within the 95% Hotelling’s *T*-squared ellipse. The results showed that the disease group had obvious metabolic disturbance compared with the control group. The performance scores of the OPLS-DA model for the day 40 data are R^2^Y = 0.96 and Q^2^ = 0.38. Similarly, the performance scores of the OPLS-DA model for the day 80 data are R^2^Y = 0.982 and Q^2^ = 0.405, which indicated that the model was stable and indicative of robust fit and prediction. To better validate the OPLS-DA model, the permutation test (*n* = 200) was used for verification. The results are shown in Figures 3C,D. The permutation test results for the R^2^Y and Q^2^ value were 0.87 and 0.38 between the control and disease groups on day 40 and 0.91 and 0.45 between the control and disease groups on day 80, respectively. The results indicated that the OPLS-DA model had no overfitting and showed good stability, making it suitable for optimization in subsequent analyses.

### Screening and Identification of Significant Differential Metabolites and Pearson’s Correlation Analysis

**FIGURE 4:**
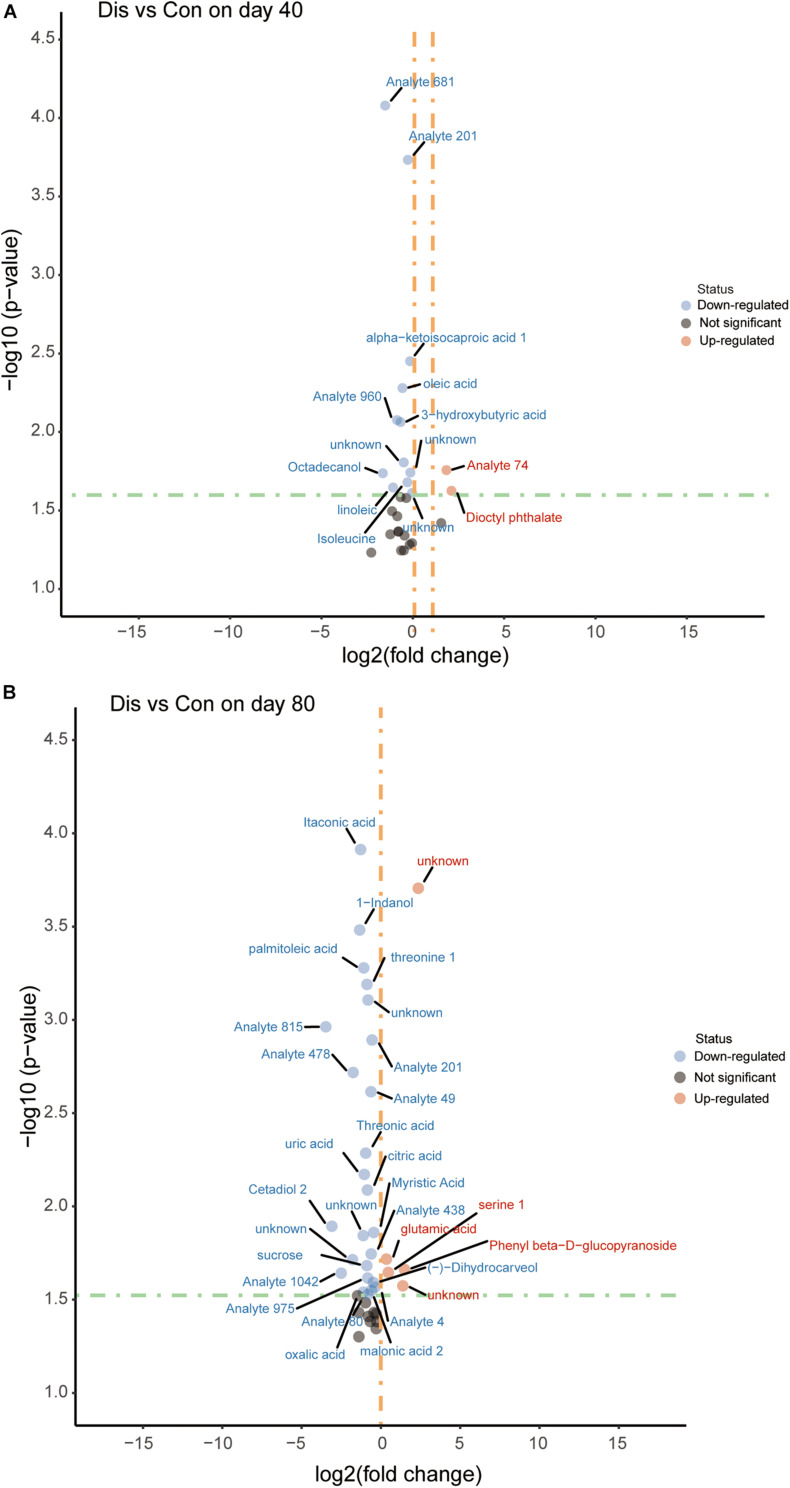
Volcano plot of serum metabolites for the disease group and the control group at days 40 **(A)** and 80 **(B)**. The x-axis represents log2 [fold change (FC)] value, and the y-axis means –log10 (*P* value). The red dots indicate that the metabolite is more abundant in the disease group, whereas blue dots indicate significantly lower metabolites compared to the control group.

Screening for significant differential metabolites was implemented in light of the VIP value (VIP > 1.0) and the Student’s *t*-test (*P* < 0.05) based on the OPLS-DA model (Wang et al., 2015). According to this analysis, it is known that metabolites are different at different time points. At day 40, 29 differential metabolites were detected and screened in the disease group compared with the control group fed with a normal diet, among which 14 metabolites were relatively quantified and 15 compounds were labeled as “analyte” or “unknown.” Similarly, on the 80th day, 40 differential metabolites were screened and identified, of which 22 compounds were relatively quantitative. The detailed information of all the differential metabolites is provided in Supplementary Tables 1, 2. The differential metabolites in the control group and the disease group were displayed in the volcano plot at the two time points (Figure 4). Compared with the control group, the levels of 3-hydroxybutyric acid, oleic acid, linoleic acid, and alpha-ketoisocaproic acid-1 in the disease group decreased significantly on the 40th day (*P* < 0.05). On the 80th day, itaconic, palmitoleic acid, threonine 1, threonic acid, citric acid, uric acid, and myristic acid in the disease group decreased significantly, while glutamic acid and serine 1 increased significantly compared with those in the control group.

**FIGURE 5:**
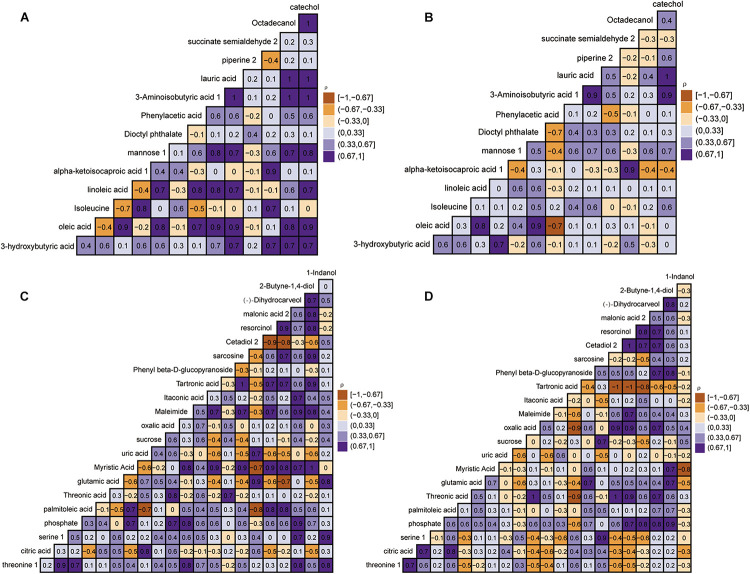
Heat map based on Pearson’s correlations between metabolites in the control group and disease group by gas chromatography time-of-flight mass spectrometry (GC-TOF-MS) analysis. The color scale represents Pearson’s correlation coefficients, with brown-yellow and purple representing negative and positive correlations, respectively. The control group at days 40 and 80 **(A,C)**. Disease group at days 40 and 80 **(B,D)**.

The relative quantitatively differential metabolites were analyzed by Pearson’s correlation coefficients to determine the correlation changes of different metabolites between the control group and the disease group. Figure 5 reveals a range of correlation coefficients between the metabolites, ranging from 1.0 (maximum positive correlation) to -1.0 (maximum anticorrelation), with 0 indicating no correlation. At day 40, the 3-hydroxybutyric was prominently and positively correlated with mannose, octadecanol, and piperine in the control group but negatively correlated with mannose, octadecanol, and piperine in the disease group. Meanwhile, isoleucine was negatively correlated with oleic acid and linoleic acid in the control group but positively correlated with oleic acid and linoleic acid in the disease group. Interestingly, at day 80, the correlation between some metabolites also showed opposite directions in the control group and the disease group. In conclusion, FLHS in laying hens induced by feeding high-energy, low-protein diets not only affected the changes of endogenous metabolites but also changed the correlations between metabolites.

### Metabolic Pathway Analysis Associated With Fatty Liver Hemorrhage Syndrome

**FIGURE 6:**
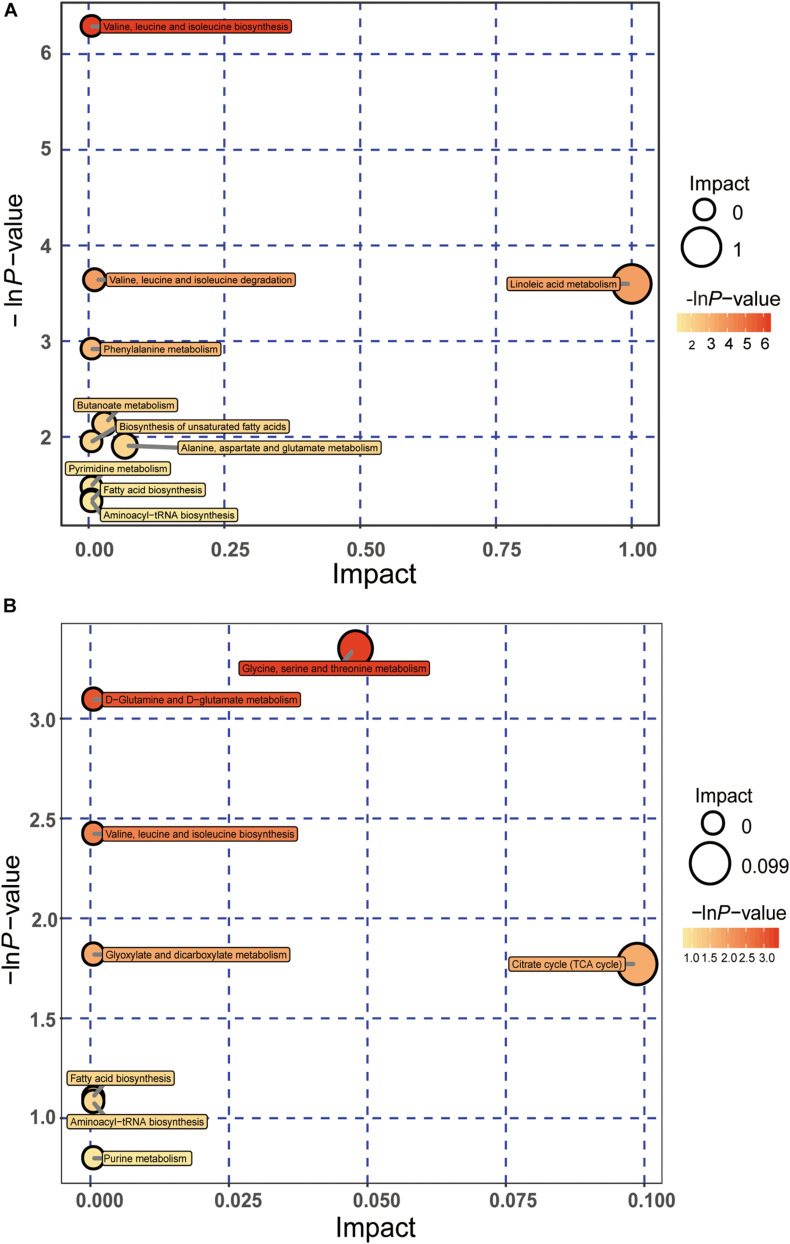
Pathway analyses of significantly differential metabolites using MetaboAnalyst, as shown in bubble plots. Bubble size is proportional to the impact of each pathway, and bubble color represents the degree of significance, from the highest (red) to the lowest (white). Panels **(A,B)** represent bubble plots enriched for metabolic pathways at days 40 and 80, respectively.

According to these significant differential metabolites, metabolomics pathway analysis was constructed to further investigate the change in metabolic pathways affected by FLHS. The results were shown by the bubble plot in Figure 6. We identified several pathways that may be significant (raw *p* < 0.5, impact > 0). The analysis showed that, at day 40, two pathways had the greatest significance: Valine, leucine, and isoleucine biosynthesis; Linoleic acid metabolism. At day 80, there were two seriously disturbed metabolic pathways, including Glycine, serine, and threonine metabolism; Citrate cycle [tricarboxylic acid (TCA) cycle]. These metabolic pathways exhibited obvious levels of dysregulation over the time course of FLHS and might facilitate FLHS development. Similarly, these pathways may also be potential targets for the formation of FLHS in laying hens.

### Acquisition of Specific Potential Biomarkers by Receiver Operating Characteristic Curve Analysis

In this study, 16 candidate biomarkers selected from significantly differential metabolites with similarity scores greater than 700 are listed in Table 1.

**TABLE 1:** Identification of significantly differential metabolites with similarity >700 in serum between the disease group and the control group at days 40 and 80.

	Metabolite	Similarity	VIP	*P* value	Fold change	Trend
Day 40	3-Hydroxybutyric acid	956	2.271	0.007	0.417	↓
	Oleic acid	931	1.018	0.004	0.449	↓
	Isoleucine	928	1.143	0.017	0.540	↓
	Linoleic acid	903	2.062	0.021	0.518	↓
	Alpha-ketoisocaproic acid 1	901	2.177	0.003	0.598	↓
	Mannose 1	850	1.500	0.036	0.283	↓
Day 80	Threonine 1	948	2.352	0.0006	0.545	↓
	Citric acid	947	2.145	0.008	0.554	↓
	Serine 1	938	1.736	0.023	1.382	↓
	Phosphate	929	1.621	0.037	0.739	↓
	Palmitoleic acid	915	2.339	0.0005	0.473	↓
	Threonic acid	911	2.243	0.005	0.5156	↓
	Glutamic acid	884	1.736	0.0192	1.272	↓
	Myristic acid	882	2.072	0.014	0.733	↓
	Uric acid	857	2.242	0.007	0.485	↓
	Sucrose	796	1.817	0.021	0.541	↓

*↑ and ↓ indicate that the metabolites increased and decreased in the disease group compared with the control group, respectively.*

**FIGURE 7:**
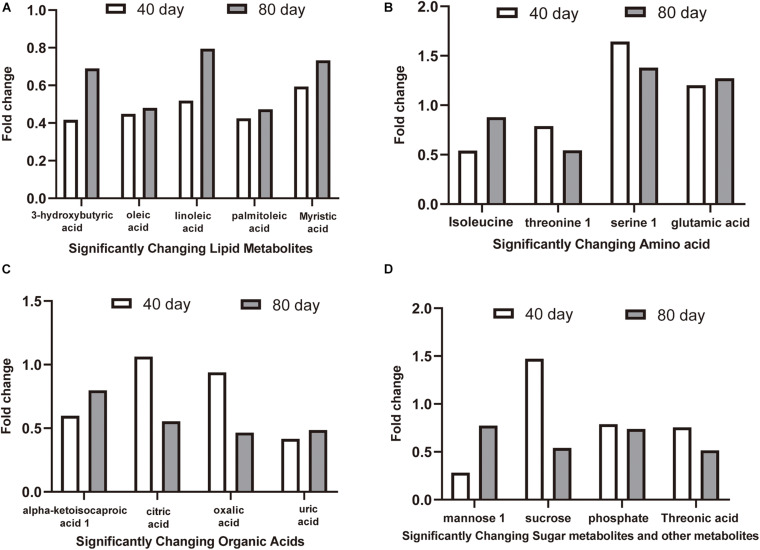
The fold change (FC) of candidate biomarkers at day 40 and day 80. FC > 1.00 indicates that the concentration of metabolites in the disease group is higher than that in the control group, and FC < 1.00 indicates that the metabolite concentration in the disease group is lower than that in the control group. **(A)** significantly changing lipid metabolites, **(B)** significantly changing amino acid metabolites, **(C)** significantly changing organic acids metabolites, **(D)** significantly changing sugar metabolites and other metabolites.

**FIGURE 8:**
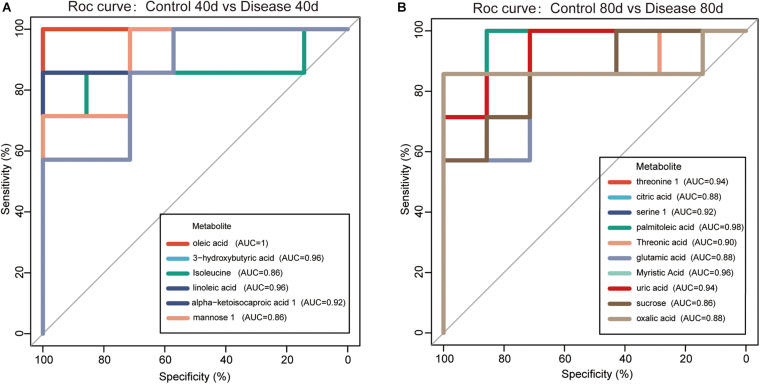
Receiver operating characteristic (ROC) curves for the diagnosis of fatty liver hemorrhage syndrome (FLHS) according to the candidate biomarkers (significant differential metabolites with similarity >700) in serum **(A)** at days 40 and **(B)** 80. The potential biomarkers were selected based on the criterion that area under the curve (AUC) values were higher than 0.9.

The 16 candidate biomarkers were divided into four categories: lipids, amino acids, organic acids, sugars, and other metabolites. The fold change (FC) of the 16 candidate biomarkers was determined at the two time points, respectively (Figure 7). Based on the FC analysis, the serum contents of differential lipid metabolites (linoleic acid, 3-hydroxybutyric acid, oleic acid, palmitoleic acid, and myristic acid), amino acid metabolites (isoleucine and threonine 1), organic acid metabolites (alpha-ketoisocaproic acid 1, uric acid, and oxalic acid), sugar, and other metabolites (mannose 1, phosphate, and threonic acid) were identified and decreased significantly at days 40 and 80. Different amino acid metabolites in the disease group such as serine 1 and glutamic acid were identified as conspicuously higher in concentration compared with the control group at days 40 and 80. Interestingly, the concentrations of citric acid and sucrose increased at day 40 (FC > 1) and decreased at day 80 (FC < 1). Then, we plotted ROC curves to clarify and estimate the prognostic performance of the candidate biomarkers and screen potential biomarkers (Figure 8). Interestingly, many candidate biomarkers showed good ROC curves with very high AUC values. Among them, 10 candidate markers possessed the top 10 AUC values (all more than 0.90), indicating an excellent discriminatory ability. The results demonstrated that oleic acid, 3-hydroxybutyric acid, linoleic acid, and palmitic acid and metabolites might be taken as potential biomarkers for diagnosing FLHS in laying hens.

## Discussion

Fatty liver hemorrhage syndrome is a nutritional and metabolic disease, which often occurs in high-yield laying hens. The main feature of the disease is lipid deposition in the liver caused by metabolic disorders, accompanied by varying degrees of bleeding, long-term fat accumulation that causes liver damage, a decrease in egg production, and even acute death of laying hens (Rozenboim et al., 2016; Shini et al., 2019). In this study, the pathological anatomy and liver tissue sections of laying hens in the disease group showed fat vacuolation and severe lipid deposition in the liver, which were consistent with our previous research results, and indicated that the model of FLHS in laying hens was successfully established. Subsequently, we applied the metabolomics method based on GC-TOF-MS to analyze the serum metabolite profile of the disease group and the control group at the two time points. A total of 40 metabolites with significant different concentrations between the control group and the disease group were screened and identified. The main metabolic pathways associated with these significantly differential metabolites are lipid metabolism, energy metabolism, and amino acid metabolism (Figure 6). We further studied these significant differential metabolites by using the ROC curves (Figure 8). The results showed that metabolites such as oleic acid, 3-hydroxybutyric acid, linoleic acid, palmitic acid, and glutamic acid showed excellent distinguishing ability and may be taken as potential biomarkers for the diagnosis of FLHS in laying hens.

Several studies have already demonstrated that 3-hydroxybutyrate was a primary ketone and a specific metabolite produced by fatty acid beta-oxidation in the liver for extrahepatic use (Newman and Verdin, 2014). The content of serum 3-hydroxybutyrate can be used as a biomarker to reflect fatty acid β-oxidation as well as ketogenic amino acid catabolism in the liver (Miyazaki et al., 2015). The results of this study suggested that the content of serum 3-hydroxybutyric acid in the disease group was significantly decreased compared to the control group at the two time points. This finding is consistent with the result of non-alcoholic fatty liver disease (NAFLD) patients with steatosis (Rodríguez-Gallego et al., 2015). Feng et al. (2019) has shown a similar result that the activation of peroxisome proliferator-activated receptor α (PPAR α) in the liver can promote the utilization of fatty acids and increase the content of 3-hydroxybutyric acid in serum. Therefore, the above results indicate the decrease of fatty acid β oxidation in FLHS laying hens.

The levels of oleic acid, linoleic acid, and palmitoleic acid were decreased in FLHS development. This result is similar to that of the study by Xu et al. (2019) on NAFLD. The significant decrease of three unsaturated fatty acids (oleic acid, linoleic acid, palmitic acid) indicates the enhancement of peroxidation and oxidative stress (Jiang et al., 2013). The enhancement of peroxidation and oxidative stress can lead to the hydrolysis of apolipoprotein B and impair the secretion of very-low-density lipoprotein (VLDL) that reduce the exports of triglycerides (TGs) from the liver and accumulates in the liver, leading to fatty liver (An et al., 2013). However, the avian ovary does not synthesize a lot of lipids, all the lipid components of the yolk are derived from plasma precursors, mainly VLDL and vitellogenin produced in the liver (Zhou et al., 2020). The hydrolysis of apolipoprotein B and the decrease of secretion of VLDL will affect the deposition of lipid nutrients in yolk and hinder the development of yolk and the occurrence of ovulation (Walzem et al., 1999; Walzem and Chen, 2014). In addition, linoleic acid is one of the essential fatty acids in the body. The lack of linoleic acid leads to a significant decline in the growth rate of animals, causing reproductive obstacle diseases and disorders of lipid metabolism in the body. Fu et al. (2020) have shown that supplementation of maternal conjugated linoleic acid (CLA) could reduce lipid synthesis through the AMP-activated protein kinase (AMPK) signal pathway in chicken embryo liver. Therefore, we speculate that the decrease of laying rate and the increase of liver fat deposition in FLHS laying hens may be related to the decrease of serum unsaturated fatty acid content.

Our findings demonstrated that serum contents of itaconic acid and saturated fatty acids (lauric acid and myristic acid) in the disease group were significantly lower than those in the control group. Itaconic acid is a substance similar to phosphoenolpyruvate. Several studies have shown that itaconic acid reduces visceral fat deposition by inhibiting hepatic glycolysis under the action of phosphofructokinase (PFK) activated by fructose 2, 6-bisphosphate (F26BP) (Sakai et al., 2004). The significant reduction of itaconic acid content in the disease group of this experiment indicated that high-energy, low-protein diets probably caused glucose metabolism disorder and fat deposition in the liver of laying hens. Lauric acid and myristic acid, as saturated fatty acids, can increase the secretion of apolipoprotein B in HepG2 cells maintained in tissue culture flasks (Arrol et al., 2000). Dietary lauric acid and myristic acid can increase the concentration of intracellular total cholesterol (Arrol et al., 2000; Gambino et al., 2016). In this study, the decrease of serum saturated fatty acids (lauric acid and myristic acid) in the disease group showed that saturated fatty acids were used for the excessive synthesis of cholesterol and secretion of apolipoprotein B was decreased in the liver, resulting in a decrease of serum saturated fatty acid content and an increase in total cholesterol. In summary, the significant fatty acid consumption indicated a decrease in fatty acid oxidation and TG release from the liver, with a consequent increase in TG synthesis, which may play an important role in the development of TG accumulation in hepatocytes (Videla et al., 2004).

As the substrate of protein synthesis, gluconeogenesis, and ketone synthesis, amino acids can also provide energy through the TCA cycle (Lopansri et al., 2006; Miao et al., 2014). Isoleucine, serine, and threonine are the precursors of succinyl coenzyme A, the intermediate metabolite of TCA cycle (Bui et al., 2019). In this experiment, the increase of serine content and the decrease of isoleucine and threonine content indicate the disorder of amino acid metabolism. Some studies have shown that mice accumulate fat through the transformation of amino acids and proteins to increase fat (Park et al., 2017). Under the influence of FLHS, laying hens may also absorb amino acids to promote fat synthesis through similar processes, which may be one of the reasons for the decrease of isoleucine and threonine. Glutamate is the precursor of α-ketoglutaric acid synthesis pathway and one of the amino acids that constitute antioxidant glutathione. The related research showed that the increase of plasma α-ketoglutarate may indicate the fatty degeneration of the liver and the potential biomarker of non-alcoholic fatty liver obesity (Rodríguez-Gallego et al., 2015). The increase of serum glutamate content in this experiment may reflect the compensatory increase of antioxidant response of FLHS induced by high-energy, low-protein diets and hepatic steatosis in laying hens. This result is consistent with a study result in which NAFLD and non-alcoholic steatohepatitis (NASH) patients with high insulin levels have significantly higher serum glutamate levels than healthy subjects (Kalhan et al., 2011).

The TCA cycle is not only the common metabolic pathway of the fat, protein, and carbohydrate but also the key link of energy metabolism *in vivo.* Citric acid is an important intermediate metabolite of the TCA cycle. In our study, the disease group showed increased citric acid at day 40 and it was significantly decreased at day 80 compared with that in the control group. One study revealed that the increase of serum citric acid can promote the biogenesis of liver mitochondria and prevent hepatic steatosis and insulin resistance (Li et al., 2016). In the study of the hyperlipidemia mouse model, the decrease of citric acid content reflected the inhibition of glycolysis and the energy metabolism dysfunction (Jiang et al., 2013). Based on these results, we speculate that, in the early stage of FLHS, the oxidative stress system is activated and the TCA cycle metabolism is increased. Over time, the accumulation of fat in the liver gradually causes further liver injury, resulting in the metabolism disorder of the TCA cycle. The disorder of TCA cycle metabolism further reflects the disorder of amino acid metabolism *in vivo.*

Mannose is a glucose-related serum metabolite mainly released by the liver and is the main source of glycoprotein synthesis (Pritchard et al., 2015; Ferrannini et al., 2020). Related studies have shown that mannose can be used as a potential biomarker of insulin resistance (Ferrannini et al., 2020). Our previous studies have also indicated that insulin resistance is highly correlated with the occurrence of FLHS in laying hens (Zhuang et al., 2019). Regrettably, the results of this study showed that mannose levels decreased in the disease group at the two time points. It is speculated that this may be related to the severity of the liver injury and the time of FLHS formation induced by high-energy, low-protein diets. Further investigation is needed to determine whether the difference in mannose is different at other time points.

## Conclusion

In summary, this study performed serum metabolomic investigations in laying hens with FLHS and provided a holistic understanding of disease progression. A total of 40 sensitive differential metabolites were identified by GC-TOF-MS-based metabolomics, which are primarily related to lipid metabolism, energy metabolism, and amino acid metabolism. Among these serum differential metabolites, 10 potential biomarkers that possess highest similarity scores and AUC values, such as oleic acid, 3-hydroxybutyric acid, linoleic acid, palmitic acid, and glutamate may be of great value for the diagnosis of FLHS.

## Data Availability Statement

The raw data supporting the conclusions of this article will be made available by the authors, without undue reservation.

## Ethics Statement

The animal study was reviewed and approved by the institutional animal care and use committee of Jiangxi Agricultural University.

## Author Contributions

XG conceived and supervised the study and acquired funding. LG, PX, CZ, YS, and JK performed the data analysis. LG, JJ, CH, and PL performed the animal study and/or contributed materials. LG and JK prepared the manuscript draft. YZ, XG, GH, and CW revised the manuscript and provided extensive discussions. All authors participated in the discussion and editing of the manuscript.

## Conflict of Interest

The authors declare that the research was conducted in the absence of any commercial or financial relationships that could be construed as a potential conflict of interest.

## Footnotes

1 http://www.genome.jp/kegg

2 http://www.metaboanalyst.ca
