# Supplementary material for: Serum Metabolomic Profiling to Reveal Potential Biomarkers for the Diagnosis of Fatty Liver Hemorrhagic Syndrome in Laying Hens
Source: Front Physiol. 2021 Feb 9;12:590638. doi: 10.3389/fphys.2021.590638 (PMC7900428; doi:10.3389/fphys.2021.590638)
Supplement: Supplementary file 1 [file Table_1.DOCX]

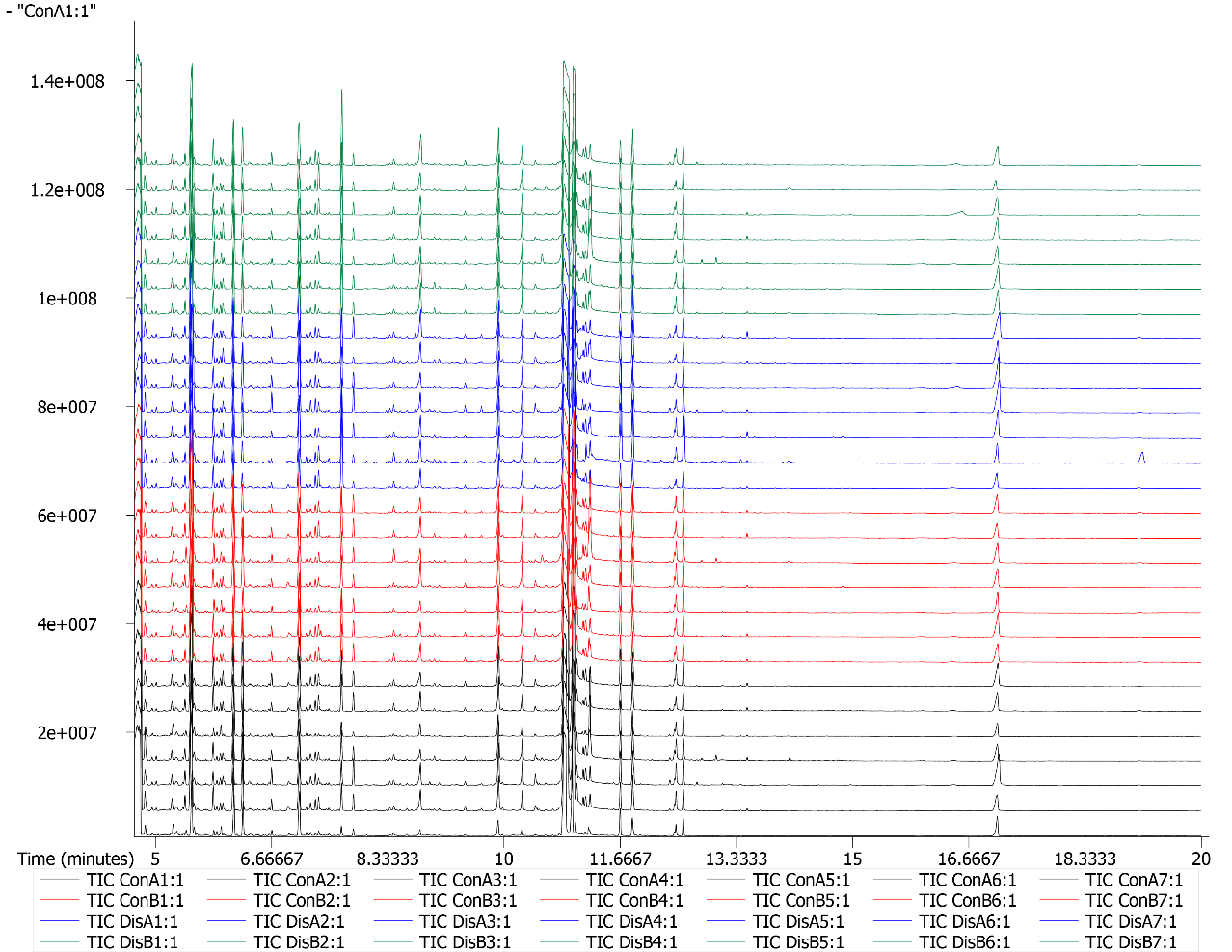


**Supplementary Figure.1** The total ion chromatogram (TIC) of the control group and the disease group was obtained by gas chromatography-time-of-flight mass spectrometry (GC-TOF-MS) in two time points. The ordinate shows the relative mass abundance, and the abscissa shows the retention time.
