# Supplementary material for: Serum Metabolomic Profiling to Reveal Potential Biomarkers for the Diagnosis of Fatty Liver Hemorrhagic Syndrome in Laying Hens
Source: Front Physiol. 2021 Feb 9;12:590638. doi: 10.3389/fphys.2021.590638 (PMC7900428; doi:10.3389/fphys.2021.590638)
Supplement: Supplementary file 2 [file Table_2.DOCX]

| Peak | Similarity | VIP | P-value | Fold change | Trend |
| --- | --- | --- | --- | --- | --- |
| 3-hydroxybutyric acid | 956 | 2.27 | 0.0069 | 0.4169 | ↓ |
| oleic acid | 931 | 1.02 | 0.0042 | 0.4488 | ↓ |
| Isoleucine | 928 | 1.14 | 0.0166 | 0.5404 | ↓ |
| linoleic acid | 903 | 2.06 | 0.0209 | 0.5184 | ↓ |
| alpha-ketoisocaproic acid 1 | 901 | 2.18 | 0.0028 | 0.5983 | ↓ |
| mannose 1 | 850 | 1.50 | 0.0356 | 0.2826 | ↓ |
| Dioctyl phthalate | 662 | 1.14 | 0.0188 | 2.8794 | ↑ |
| Phenylacetic acid | 634 | 1.45 | 0.0254 | 0.3019 | ↓ |
| 3-Aminoisobutyric acid 1 | 539 | 1.29 | 0.0414 | 0.5895 | ↓ |
| unknown | 508 | 1.19 | 0.030 | 1.9477 | ↑ |
| lauric acid | 505 | 1.63 | 0.0451 | 0.4725 | ↓ |
| piperine 2 | 312 | 1.20 | 0.0273 | 0.3684 | ↓ |
| succinate semialdehyde 2 | 298 | 1.78 | 0.0405 | 0.6444 | ↓ |
| Octadecanol | 272 | 2.14 | 0.0145 | 0.2126 | ↓ |
| catechol | 261 | 1.65 | 0.0363 | 0.4848 | ↓ |
| unknown | 0 | 1.68 | 0.0140 | 2.3792 | ↑ |

**Supplementary Table.S1** The potential metabolites detected by the GC-TOF-MS analysis were compared between the disease group and the control group at 40 days. ↑ and ↓ indicate that the metabolites increased and decrease in the disease group than the control group, respectively.
