# Supplementary material for: Serum Metabolomic Profiling to Reveal Potential Biomarkers for the Diagnosis of Fatty Liver Hemorrhagic Syndrome in Laying Hens
Source: Front Physiol. 2021 Feb 9;12:590638. doi: 10.3389/fphys.2021.590638 (PMC7900428; doi:10.3389/fphys.2021.590638)
Supplement: Supplementary file 3 [file Table_3.DOCX]

| Peak | Similarity | VIP | P-value | Fold change | Trend |
| --- | --- | --- | --- | --- | --- |
| threonine 1 | 948 | 2.35 | 0.0006 | 0.5446 | ↓ |
| citric acid | 947 | 2.15 | 0.0082 | 0.5544 | ↓ |
| serine 1 | 938 | 1.74 | 0.0226 | 1.3816 | ↑ |
| phosphate | 929 | 1.62 | 0.0371 | 0.7394 | ↓ |
| palmitoleic acid | 915 | 2.34 | 0.0005 | 0.4731 | ↓ |
| Threonic acid | 911 | 2.24 | 0.0052 | 0.5156 | ↓ |
| glutamic acid | 884 | 1.74 | 0.0192 | 1.2724 | ↑ |
| Myristic Acid | 882 | 2.07 | 0.0138 | 0.7325 | ↓ |
| uric acid | 857 | 2.24 | 0.0067 | 0.4854 | ↓ |
| sucrose | 796 | 1.82 | 0.0208 | 0.5413 | ↓ |
| oxalic acid | 765 | 1.13 | 0.0288 | 0.4655 | ↓ |
| Maleimide | 610 | 1.28 | 00371 | 0.3795 | ↓ |
| Itaconic acid | 561 | 2.20 | 0.0001 | 0.4130 | ↓ |
| unknown | 505 | 1.86 | 0.0266 | 2.6086 | ↑ |
| Tartronic acid | 423 | 1.04 | 0.0389 | 0.5800 | ↓ |
| Phenyl beta-D-glucopyranoside | 405 | 1.75 | 0.0218 | 2.8113 | ↑ |
| sarcosine | 379 | 1.72 | 0.0415 | 0.8388 | ↓ |
| Cetadiol 2 | 370 | 1.77 | 0.0128 | 0.1176 | ↓ |
| unknown | 324 | 2.24 | 0.0002 | 5.1263 | ↑ |
| resorcinol | 310 | 1.77 | 0.0328 | 0.5104 | ↓ |
| malonic acid 2 | 291 | 1.69 | 0.0282 | 0.6631 | ↓ |
| (-)-Dihydrocarveol | 288 | 1.77 | 0.0256 | 0.7201 | ↓ |
| 2-Butyne-1,4-diol | 278 | 1.62 | 0.0376 | 0.8152 | ↓ |
| 1-Indanol | 263 | 1.27 | 0.0003 | 0.3960 | ↓ |

**Supplementary Table.S2** The significant difference metabolites detected by the GC-TOF-MS analysis were compared between the disease group and the control group at 80 days. ↑ and ↓ indicate that the metabolites increased and decrease in the disease group than the control group, respectively.
